# Detection of exogenous sugars in pineapple juice using compound-specific stable hydrogen isotope analysis

**DOI:** 10.1038/s41538-021-00092-5

**Published:** 2021-04-14

**Authors:** Simon D. Kelly, Aiman Abrahim, Peter Rinke, Andrew Cannavan

**Affiliations:** 1 Food and Environmental Protection Laboratory, Joint FAO/IAEA Centre of Nuclear Techniques in Food and Agriculture, Department of Nuclear Sciences and Applications, International Atomic Energy Agency, Vienna International Centre, Vienna, Austria; 2 SGF International e.V., Marie-Curie-Ring 10a, Saulheim, Germany

**Keywords:** Sustainability, Dietary carbohydrates

## Abstract

An improved procedure for determining ^2^H/^1^H isotope ratios, using gas chromatography-isotope ratio mass spectrometry, has been used to detect the addition of exogenous C4-plant-derived sugars to pineapple juice. Isotopic techniques are commonly used to identify the addition of low-cost sugars to fruit juices and are difficult to subvert as it is not economically viable to change the isotopic ratios of the sugars. However, the addition of cane sugar to pineapple juice has presented a significant challenge that is only detected by site-specific ^13^C analysis of the methyl and methylene positions of ethanol derived from pineapple sugars, measured by nuclear magnetic resonance. This new GC-IRMS-based procedure utilises the trifluoroacetate derivative of sucrose to allow direct measurement of the carbon-bound non-exchangeable hydrogen. This provides advantages over alternative isotopic methods in terms of analysis time and sensitivity. This feasibility study has demonstrated the potential to reliably differentiate between authentic pineapple juices and those adulterated with commercial beet and cane sucrose.

## Introduction

Stable isotope analysis has been effectively used to detect the economically motivated adulteration of fruit juices for decades. This has involved both the use of gas isotope ratio mass spectrometric analysis, for example, carbon and oxygen isotopes in the soluble solids and water in fruit juice, respectively, and site-specific deuterium/hydrogen ratios (^2^H/^1^H) by quantitative deuterium nuclear magnetic resonance of ethanol fermented from fruit juice sugars^1,2^. Over the years, methods have been improved and refined with the introduction of continuous-flow IRMS measurements of ^2^H/^1^H^3^. These methods now form an important part of the Quality Systems used by fruit juice manufacturers worldwide and are listed in the Codex Alimentarius Standard 234^4^, and recognised by the AOAC^5,6^. The detection of low-value sugar syrups derived from sugar cane and corn starch is routinely achieved using stable carbon isotope analysis. Differences in the photosynthetic pathway utilised by corn and cane plants (Hatch-Slack, C4) to fix carbon dioxide and the majority of other plant species (Calvin, C3) permit the presence of the adulterant sugar syrups to be detected in fruit juices^7^, maple syrup^8^ and honey^9^. These plant metabolic differences typically result in δ^13^C values between −10‰ and −12‰ for corn and cane sugars and syrups therefrom, whereas the majority of other important agricultural crops typically possess δ^13^C values between −23‰ and −28‰^10^. Consequently, the addition of C4 sugar syrup to fruit juices, for example, produces a deviation from the normal δ^13^C value expected for a C3 plant-derived food product, which can be detected using carbon stable isotope ratio analysis. Detection limits of C4 adulterant carbohydrates can be further improved by using internal isotopic correlations with other unadulterated components in the food such as pulp in fruit juice^11^ or other related sugar correlations e.g. between individual carbohydrates using liquid chromatography–isotope ratio mass spectrometry^12^. However, the major disadvantage of carbon stable isotope analysis is that it cannot be used to detect the addition of a C3 beet sugar syrup to a C3 food product such as orange juice, especially if the sugar profile is carefully matched (e.g. sucrose, glucose and fructose ratio) and other ingredients such as citric acid, l-malic acid and vegetable–water are added to mask dilution, for targeted analysis, and restore the sugar:acid ratio. However, the addition of commercial sweeteners such as beet medium invert syrup (BMIS) can be detected by isotope ratio mass spectrometry if the deuterium (^2^H)/protium (^1^H) isotope ratio of the carbon-bound non-exchangeable (CBNE) hydrogen atoms in the sugars is measured. The natural variation in ^2^H/^1^H ratios varies significantly in biogeochemical cycles due to the large relative difference between the mass of the two stable isotopes of hydrogen. Consequently, the fractionation effects are generally more manifest for hydrogen than for the heavier bioelements such as carbon, nitrogen, oxygen and sulfur. In general, the deuterium content of precipitation decreases with increasing latitude, altitude and distance from the sea. This systematic variation is translocated into plant tissues that are cultivated in the corresponding locations and reflected in products such as beet sugar, which is normally grown further from the equator in more temperate regions, compared with citrus and tropical fruits, and therefore contains less deuterium than the sugars present in many fruit juice crops such as orange and pineapple. Furthermore, differences in plant morphology and the relative rates of leaf-stomatal evapotranspiration between aerial plants such as fruit trees and ground plants such as the beet-sugar plant, which has a relatively small surface area of leaves, result in a significant difference in the ^2^H/^1^H ratio of leaf water prior to assimilation and the consequent *δ*^2^H values of carbon-bound hydrogen of organic plant components in these species. These differences in *δ*^2^H values can be used to detect the addition of C3 plant sugars or syrups from beet sucrose to C3 fruit juices such as orange juice^13^.

However, there has been a deficiency in these widely applied fruit juice authenticity isotopic methods. Pineapple juice sugar global *δ*^13^C values, and site-specific SNIF-NMR (^2^H/^1^H)_1_ measurement of ethanol therefrom, overlap with sugars derived from cane and maize (C4 plants). According to the definitions of the consolidated EC fruit juice directive 2012/12^14^ and the AIJN code of practice guidelines^15^, pineapple juice must be extracted from the edible part of *Ananas comosus (L.) Merr*. If sugar or sweeteners are added, the resulting product should be declared with another denomination, e.g. pineapple ‘nectar’ or ‘drink’. Pineapple juice is unique in that it is the only fruit juice widely consumed that is produced by a plant utilising the crassulacean acid metabolism (CAM) to fix carbon dioxide and water to produce plant components. The global *δ*^13^C values and SNIF-NMR (^2^H/^1^H)_1_ values of pineapple, cane and maize sugars overlap to such an extent that they cannot reliably detect their addition in isolation or when masked by other adulterations. This deficiency in the ^2^H SNIF-NMR technique may be attributed to the loss of pertinent information from non-exchangeable hydrogen in the sugars during their conversion to ethanol and the site-specific nature of the information derived from the 1, 6 and 6’ positions of glucose^16^. Similarly, the climatic conditions under which pineapples are cultivated mean that the CO_2_ fractionation associated with the CAM photosynthetic pathway tends towards that of the C4-plant metabolism producing similar global *δ*^13^C values. However, this deficiency was overcome in 2010 by the development of ^13^C SNIF-NMR analysis (site-specific ^13^C/^12^C ratio measurements) of the methyl and methylene positions of ethanol derived from pineapple juice sugars with a detection limit of C4 sugars around 15% of the total present^17^. Nevertheless, the facts remain that this method is time consuming and relatively expensive to implement due to the requirement to undertake a minimum 3-day fermentation of the pineapple juice sugars to ethanol, perform a near-quantitative distillation to recover ethanol without isotopic fractionation and the measurement with a high-field NMR (typically 400-MHz proton or higher). Recently, Abrahim, Cannavan and Kelly reported the use of a volatile fluorinated derivative of carbohydrate facilitating the rapid *δ*^2^H measurement of carbon-bound non-exchangeable (CBNE) hydrogen by compound-specific stable isotope analysis (CSIA)^18^. This permits the global hydrogen isotopic composition of individual mono- and disaccharides to be reliably measured with a repeatability of less than 3‰ (σn−1). In this paper, we describe the application of that method to detect the economically motivated adulteration of pineapple juice, with results elucidated within 48 h. The procedure utilises a simple and convenient one-step reaction to substitute the exchangeable hydroxyl-hydrogens on sugars from pineapple juice with trifluoroacetate (TFA) derivatives that are sufficiently volatile to be separated by gas chromatography and measured by an isotope ratio mass spectrometer coupled to a gas chromatograph. The derivatised sugars are converted into hydrogen gas using a high-temperature chromium-silver reactor that retains carbon, oxygen and fluorine, whilst the mass distribution of unretained hydrogen isotopologues is measured by gas isotope ratio mass spectrometry. This new procedure has advantages over existing methods in terms of ease of use, analysis time and compound-specific information. The *δ*^2^H of CBNE hydrogen atoms from sucrose, measured by gas chromatography–chromium silver reduction/high-temperature conversion–isotope ratio mass spectrometry (GC–CrAg/HTC–IRMS), therefore provides a new possibility for differentiating between the botanical origin of pineapple and sugar cane carbohydrate.

## Results

### Quality control of the isotope analyses

The repeatability of the CBNE *δ*^2^H measurement of the trifluoracetate derivative of sucrose by GC–Cr/HTC–IRMS was previously reported by Abrahim et al. as ± 2.0‰ (σn−1, *n* = 18, over 3 days)^18^, which is in line with expectations for compound-specific hydrogen stable isotope analysis through a GC inlet ≤3‰^19^. In this study, isolated pineapple juice sugars were derivatized and analysed in triplicate and the sample standard deviation (σn−1, *n* = 3) calculated as a measure of individual pineapple juice sucrose CBNE *δ*^2^H measurement precision. The measurement precision for the 20 authentic pineapple juices reported in this study ranged from 0.2 to 2.2‰ with an average value of 1.1‰ (see Supplementary Data). Similarly, the bulk *δ*^13^C measurement of the sugars isolated from the 20 authentic pineapple juices was measured in triplicate by EA-IRMS and the precision (σn−1, *n* = 3) ranged from 0.02 to 0.40‰ with an average value of 0.12‰ (see Supplementary Data).

### *δ*^2^H and values derived from the non-exchangeable hydrogen and bulk *δ*^13^C from carbon present in the sugars from authentic pineapple juice

**Fig. 1:**
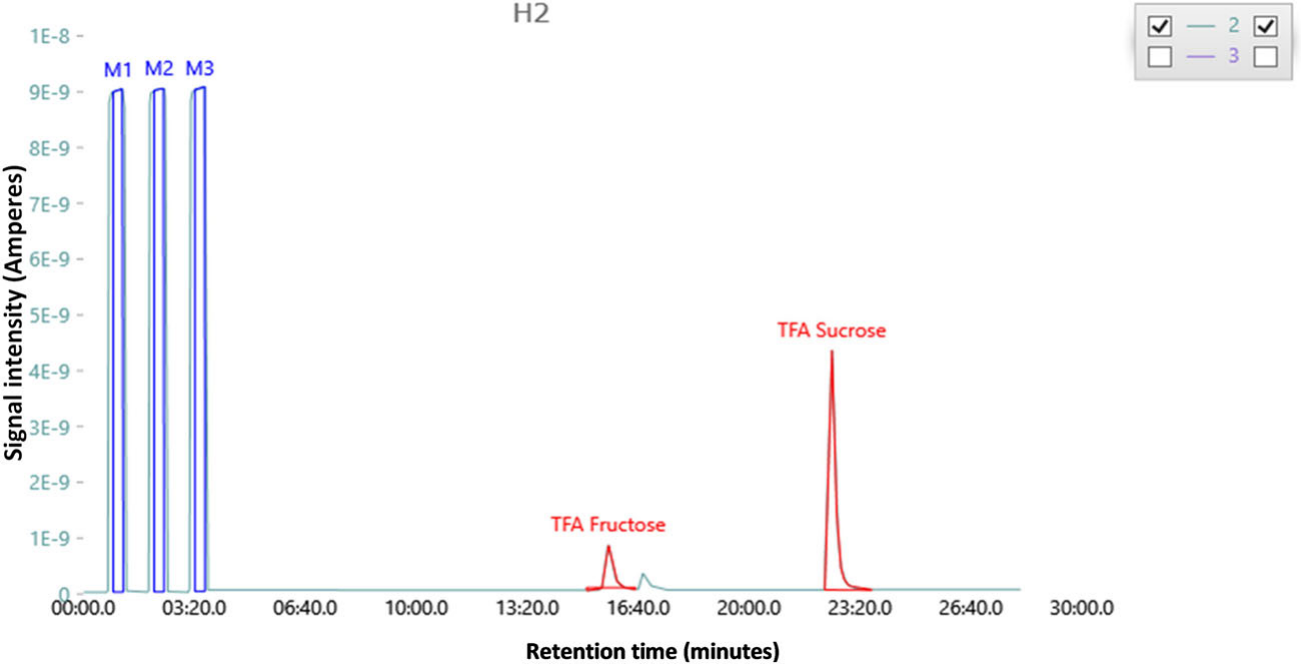
Ion chromatogram of a sugar extract from authentic pineapple juice. The *m/z* 2 trace shows the sugars as their corresponding trifluoroacetate (TFA) derivatives, analysed by GC–CrAg/HTC–IRMS.

A typical *m/z* 2 ion chromatogram of a sugar extract from authentic pineapple juice, as its corresponding trifluoroacetate (TFA) derivative, measured by GC–CrAg/HTC–IRMS, is shown in Fig. 1. The *δ*^2^H of the sucrose CBNE hydrogen and *δ*^13^C values of the total sugars prepared from twenty authentic commercial pineapple juices are summarised in Table 1. In addition, Table 1 contains the data obtained from commercial samples of sugar beet and sugarcane sucrose. The pineapple juice sucrose CBNE *δ*^2^H and bulk sugar *δ*^13^C results are grouped by country of origin and include the individual country and all countries: mean, SD, maximum and minimum values. Sucrose was selected because it was generally found to be the highest-concentration sugar present in pineapple juice and provides the highest-intensity peaks for repeatable *δ*^2^H measurement. Moreover, the cheapest common adulterant in pineapple juice is cane sugar sucrose.

**Table 1:** The *δ*^2^H of the sucrose CBNE hydrogen and *δ*^13^C values of the total-sugars prepared from authentic commercial pineapple juices and from commercial samples of sugar beet and sugar cane sucrose.

				Sucrose CBNE *δ*^2^H [‰] by GC-CrAg/HTC-IRMS					Total sugar (bulk) *δ*^13^C [‰] by EA-IRMS			
Country of origin	Sample type	Year	*n*	Mean	Maximum	Minimum	SD (σn−1)	*n*	Mean	Maximum	Minimum	SD (σn−1)
Brazil	CPJ, PJP		4	12.2	24.2	−8.1	14.2	4	−13.56	−12.64	−14.28	0.81
China	SSPJ		1	12.7	12.7	12.7	na	1	−13.04	−13.04	−13.04	na
Costa Rica	SSPJ		1	7.5	7.5	7.5	na	1	−13.32	−13.32	−13.32	na
Indonesia	CPJ		2	−3.6	2.6	−9.9	na	2	−12.88	−12.8	−13.0	na
Kenya	PJP		1	17.3	17.3	17.3	na	1	−12.52	−12.52	−12.52	na
Philippines	CPJ		1	−25.0	−25.0	−25.0	na	1	−13.37	−13.37	−13.37	na
South Africa	CPJ		1	16.0	16.0	16.0	na	1	−11.29	−11.29	−11.29	na
Thailand	CPJ		8	−7.4	31.2	−39.6	22.3	8	−13.5	−12.9	−14.9	0.6
Vietnam	SSPJ		1	−4.5	−4.5	−4.5	na	1	−14.08	−14.08	−14.08	na
**All countries**	**SSPJ, CPJ, PJP**		**20**	**0.34**	**31.2**	**−39.6**	**18.7**	**20**	**−13.29**	**−11.29**	**−14.87**	**0.76**
Cane sugar	Sucrose		5	−84.2	−55.0	−101.0	17.6	3	−11.66	−10.80	−12.26	0.76
Beet sugar	Sucrose		3	−157.9	−145.6	−169.6	12.0	3	−26.80	−26.13	−27.21	0.58

*CPJ* concentrated pineapple juice, *PJP* pineapple puree, *SSPJ* single strength pineapple juice, *na* not applicable.

## Discussion

The bulk *δ*^13^C values of the total sugars generally fall in line with those in the ‘Reference Guideline for Pineapple Juice’ between −13.5 and −11.0‰ reported by AIJN—European Fruit Juice Association^20^. In some cases, sugar *δ*^13^C values have been measured outside this range and this is attributed to the pineapple plant’s Crassulacean Acid Metabolism (CAM) adapting to its local environment to a greater extent than plants with C3 or C4 metabolism. As expected, beet sugar is easily distinguished from pineapple juice based on carbon isotope stable ratios, with the mean *δ*^13^C value for the beet sucroses at −26.80‰ and the overall mean value for the authentic pineapple juices’ *δ*^13^C value at −13.29‰. However, the range of the majority of cane sugar *δ*^13^C values (−10.80‰ to −12.26‰) lies within the range of values for authentic pineapple juice sugars (−11.29‰ to −14.87‰). Furthermore, the pineapple juice values correspond well with previously reported data^21^ and are in line with the guidance values from the European Fruit Juice Association with the exception of the Vietnamese sample at −14.08‰. There is much more variation in the CBNE hydrogen isotope values of the pineapple juice due to the relative mass difference in the isotopes of hydrogen, leading to greater mass-dependent fractionation during incorporation of leaf-water into sugars during photosynthesis and subsequent conversion to disaccharides. The CBNE *δ*^2^H values vary from −39.6 to 31.2‰ (*n* = 8) for samples from Thailand and this range encompasses all other authentic pineapple juice sample values. There are limited samples for other countries of origin, and therefore the true extent of natural variation in the CBNE hydrogen isotope values will require further sampling of authentic industrially produced pineapple juices. The ‘geographical influence' on the hydrogen isotopic composition of agricultural products is well documented^22^. Hydrogen isotopes in water vary systematically across the globe due to fractionating processes in the hydrological cycle^23^. Plants assimilate the isotopologues of water through photosynthesis and incorporate them into plant components with frequently consistent offsets, for example, due to leaf-water evapotranspiration prior to assimilation. Consequently, products such as apple juice, which is produced from fruit cultivated in both relatively hot and temperate climates, over a wide range of latitudes, display an observable correlation between the latitude of the country of production and the CBNE hydrogen isotope values as reported previously for European, New Zealand, Australian and South African apples where a general increase in the ^2^H/^1^H ratio was observed with decreasing latitude of the country of origin^24^. However, the authentic pineapple juices reported here were all produced within a relatively narrow range of tropical latitudes (± 23°27’ North) apart from one sample from South Africa produced at a latitude of −33°01’ North and do not display a significant correlation between the latitude of production and sucrose CBNE *δ*^2^H values. Furthermore, irrigation crops with water derived from remote sources may also confound expected hydrogen isotope correlations and this was observed previously for apples irrigated with Cascade mountain water in State in the USA. Using the ‘Online Isotopes in Precipitation Calculator’ (OIPC), it is possible to estimate the mean annual *δ*^2^H values of precipitation in the production areas of the pineapple juices^25–27^. From this, an estimate of the enrichment in sucrose CBNE ^2^H can be calculated, and it was found that on average, the ^2^H content of CBNE hydrogen in sucrose was enriched relative to the mean annual local meteoric water by 32‰ and by as much as 75‰.

The data from this feasibility study shown in Table 1 indicate that on average, the addition of exogenous C3- (*δ*^2^H = −157.9 ± 12.0‰) or C4- (*δ*^2^H = −84.2 ± 17.6‰) derived sucrose to pineapple juice (*δ*^2^H = +0.34 ± 18.7‰) may be detected on the basis of stable isotope analysis of the non-exchangeable hydrogen using the derivatization- and compound-specific stable isotope analysis method described here. This finding is in agreement with previously reported work on the hydrogen isotopic composition of plant carbohydrate, i.e. that it can be used to distinguish between plants using the CAM photosynthetic pathway (*δ*^2^H = +51 ± 10‰) from those using C3 and C4 pathways (*δ*^2^H = –40 ± 20‰) according to Sternberg et al.^28^. Although the data are limited in this feasibility study, the mean values and standard deviations for the authentic pineapple juice and cane sugar CBNE *δ*^2^H datasets can be used to calculate probability densities of the Gaussian, or normal, distribution curves for the entire populations of each group, assuming that they follow such a distribution. Equation 1 is used to calculate the normal distribution:

1 $$y = \frac{1}{{\sigma \sqrt {2\pi } }}e^{ - [\frac{{\left( {x - \mu } \right)^2}}{{2\sigma ^2}}]}$$

where *y* = frequency, *x* = CBNE *δ*^2^H value, *µ* = population mean and *σ* = population standard deviation. The values of *µ* and *σ* are not known, but *x* and *σ*^n−1^ may be used as estimates from the values derived from the authentic samples. *z*-scores of 1.96 and 2.58 are the limits on either side of a normal distribution population mean in which 95 and 99% of all observations will lie, respectively. In this feasibility study with limited sample numbers, the uncertainty in the estimates of *µ* and *σ* must be compensated for by increasing the values of 1.96 and 2.58 accordingly from the mean, and the symbol *z* is replaced by *t*. *t*-distributions are determined, not only by the mean and standard deviation but also taking into consideration the size of the population (i.e. the number of observations in the sample, *n*). Critical *t*-scores, at different confidence intervals, may be found in statistical tables, for example in Miller and Miller^29^. The *t*-score for an observation *x* can be calculated using Eq. 2:

2 $$t = \frac{{x - \bar x}}{{\sigma^{{\rm{n}} - 1}}}$$

where *x* = value of an individual observation (e.g. pineapple juice sucrose CBNE *δ*^2^H value), *x* = mean of the sample set and *σ*^n−1^ = standard deviation of the authentic sample set. For any given observation with a specific CBNE *δ*^2^H value (*x*), if the calculated absolute value of *t* is greater than the critical value of | *t* | given in the tables for (*n* − 1) degrees of freedom *Df* at a confidence interval (CI) of 95% (*P* = 0.05), it can be concluded that the observation is ‘unlikely' to come from the same population, with the same mean and standard deviation, as the authentic pineapple dataset. Similarly, if the calculated value of *t* is greater than *t*-critical, for (*n* − 1) degrees of freedom at a confidence interval of 99% (*P* = 0.01), then it can be concluded that the observation is ‘highly unlikely' to have come from the same population as that from which the sample was drawn^30^. In this study, the critical *t*-values based on 20 authentic pineapple juices (*Df* = 19), obtained by interpolation between the values given in standard statistical tables, are 2.095 at a CI of 95% and 2.865 at a CI of 99%. These *t*-values can be substituted into Eq. 2, with the corresponding *x* and *σ*^n−1^ values, to calculate the values of *x* (sucrose CBNE *δ*^2^H) within which 95 and 99% of all authentic pineapple juice observations are expected to lie.

**Fig. 2:**
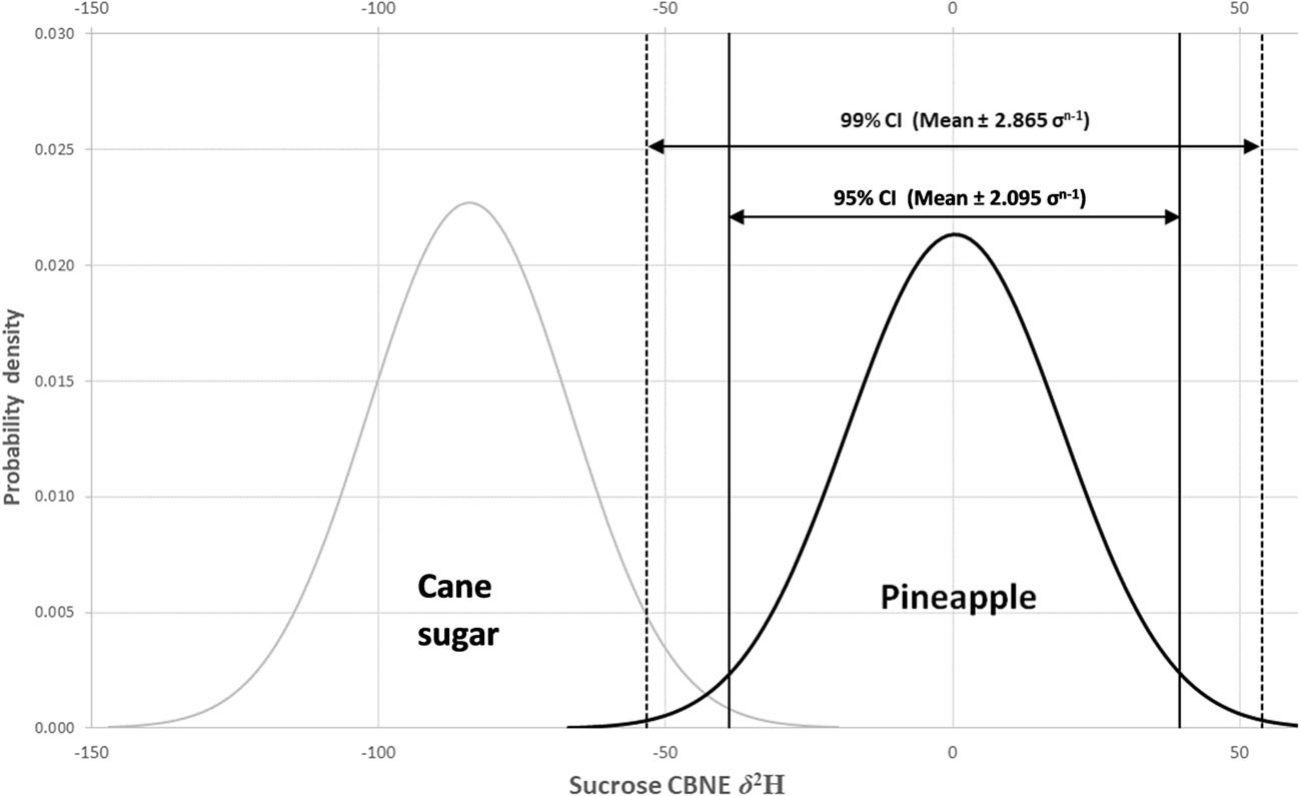
Gaussian (or normal) distributions created using the NORMDIST function in Microsoft Excel. The distributions are generated from specified means and standard deviations, for pineapple (black curve) and cane sugar (grey curve) sucrose CBNE *δ*^2^H values. The solid and dashed vertical lines show the limits between which 95 and 99% of the authentic pineapple juice population would be expected to fall, respectively. These boundaries are based on the mean and standard deviation of the measured dataset and assume that the measured dataset is representative of the entire pineapple juice population and it follows a normal distribution.

Normal distribution curves for the authentic pineapple juice and sugar cane sucrose CBNE *δ*^2^H populations, created using the Microsoft Excel *NORMDIST* function, are shown in Fig. 2. Ninety-five percent (*P* = 0.05) of the calculated authentic pineapple juice population would be expected to have sucrose CBNE *δ*^2^H isotope values between −38.9 and 39.5‰. The remaining 5% of the pineapple juice population would be expected to be equally distributed between the two curve-tails i.e. 2.5% each. Consequently, 5 out of 200 authentic pineapple juice samples could be expected to have sucrose CBNE *δ*^2^H values less than −38.9‰ and only 5 out of 200 to have values greater than 39.5‰. Pineapple juices with sucrose CBNE *δ*^2^H values less than −38.9‰ and greater than 39.5‰ may be described as ‘statistically unlikely’ to come from a population with the same mean as the authentic pineapple juices analysed during this feasibility study.

Similarly, pineapple juices with sucrose CBNE *δ*^2^H values of less than −53.3‰ or greater than 54.0‰ are statistically ‘highly unlikely’ to come from an authentic pineapple population, with the same mean, as those analysed during this feasibility study. In this situation, only 1% of the entire modelled pineapple juice population would be expected to lie in each of tails, either side of the normal distribution (*P* = 0.01) i.e. 1 out of 200 authentic pineapple juice samples would be expected to have a sucrose CBNE *δ*^2^H value less than −53.3‰ and only 1 out of 200 to have a value greater than 54.0‰. In this feasibility study, there is a slight overlap between the sugarcane sucrose CBNE *δ*^2^H values, ranging between −101.0 and −55.0‰, and the modelled pineapple juice sucrose 99% CI minimum value of −53.3‰. It should be noted that although the number of cane sugar samples is limited (*n* = 5), there is good agreement with the range of cane sucrose CBNE *δ*^2^H values reported in previous studies, using dual-inlet IRMS measurement of offline combusted sugar-nitrate esters of −74.1^31^, −63^31^ and −41‰^32^. In principle, these values should be comparable with the CBNE *δ*^2^H values of sugarcane sucrose-TFA derivatives measured by GC–CrAg/HTC–IRMS, meaning that the population range for cane sugar is wider than the samples measured here. Furthermore, the interpretation of the pineapple juice sucrose CBNE *δ*^2^H data, as described above, assumes that the pineapple dataset is representative of the corresponding entire population and is normally distributed, i.e. that these authentic sample ‘subsets’ of the entire population are unbiased in any way. In fact, they do contain bias, as the authentic pineapple juice samples were dominated by those cultivated in Thailand, and consequently these data are not a random subset of the entire pineapple juice population. At the same time, this subset does include at least one pineapple juice sample from each of the typical producing countries in the world: Brazil, China, Costa Rica, Indonesia, Kenya, Philippines, South Africa, Thailand and Vietnam.

**Fig. 3:**
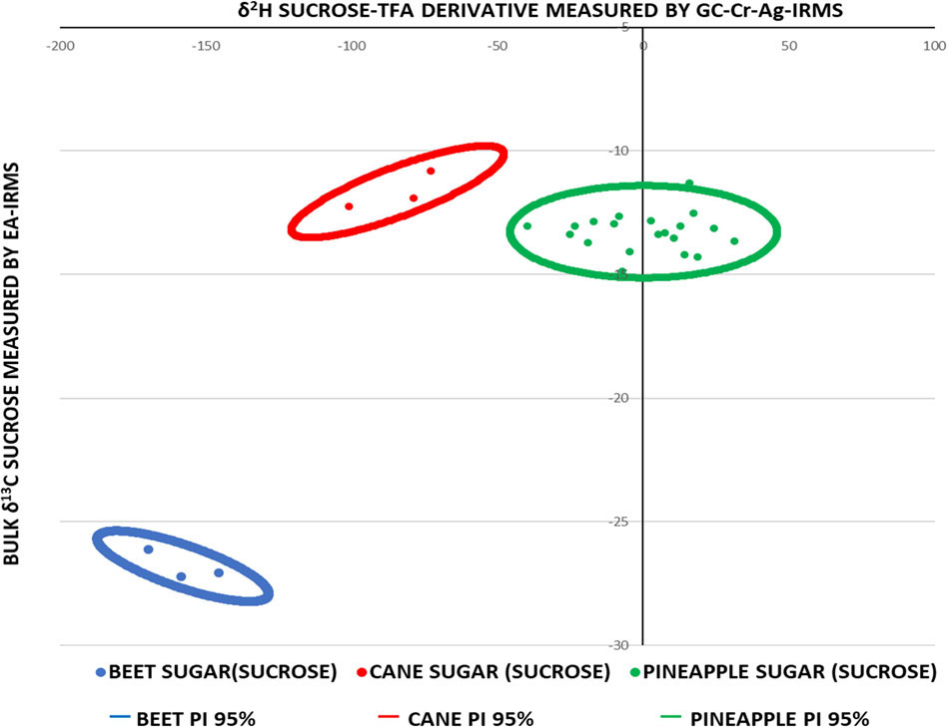
Plot of authentic pineapple juice, beet and cane sucrose-TFA derivative *δ*^2^H values versus the corresponding bulk sugar *δ*^13^C values. In all, 95% prediction interval (PI) ellipses are also shown for each group.

Further bivariate elaboration of the data is shown in Fig. 3. Here, the 95% prediction ellipses have been calculated from the bulk δ^13^C analysis of lyophilised sugars extracted from pineapple juice and the corresponding CBNE *δ*^2^H values of the sucrose-TFA derivative using Microsoft Excel (for office 365 MSO)^33^. A prediction ellipse is a bivariate region for predicting a new observation in the population. It also approximates a region that contains a specified percentage of the population, in this case 95% (*P* = 0.05), given that the new observation comes from a bivariate normal distribution. Consequently, the prediction ellipses may be used to assess the authenticity of samples with respect to the addition of either exogenous C4 or C3 sugars e.g. cane and beet sugar (syrups), respectively. The authors acknowledge that the likelihood of C3 sucrose addition is low due to its relatively straightforward detection by bulk *δ*^13^C analysis with EA-IRMS.

In order to demonstrate that there was a linear relationship between pineapple sucrose CBNE *δ*^2^H values and those obtained when increasing amounts of cane sugar are added, we previously reported the analysis of a freshly squeezed pineapple juice with a sucrose CBNE *δ*^2^H values +14.17 ± 0.69‰ (*n* = 4) with which we simulated an adulteration with cane sugar sucrose, with a CBNE *δ*^2^H value of −101.12 ± 2.8‰ (*n* = 4)^18^. The cane sucrose was added at 10% w/w and 20% w/w of the sucrose present and the corresponding CBNE *δ*^2^H values were determined. The correlation coefficient (*R*^2^) between the measured and calculated sucrose *δ*^2^H values for 0, 10, 20 and 100% w/w cane sugar in the freshly squeezed pineapple juice was 0.9997, with a slope of 1.0040 and an intercept of +0.6‰. This experiment demonstrated the linear mixing relationship for exogenous cane (C4) sucrose to pineapple sucrose and measured CBNE *δ*^2^H values. Furthermore, we previously reported the analysis of a retail sample labelled as a ‘50% pineapple juice’ drink^18^. The addition of exogenous sugars and citric acid was declared on the label and the measured CBNE *δ*^2^H values were −86.3 ± 1.5‰ (*n* = 3). This value is, as expected, 30‰ lower than the −53.3‰ cut-off value for the 99% confidence interval and would be regarded as statistically ‘highly unlikely’ to come from an authentic pineapple population with the same mean as those analysed during this feasibility study. In its current stage of development, this methodology offers an alternative and reliable means of detecting adulteration of pineapple juice with commercial beet- and cane-derived sucrose using stable isotope analysis of CBNE hydrogen. To further improve the detection limit of the quantity of added cane sugar, an internal or intermolecular isotopic reference may be used to improve the sensitivity of the technique. This potential improvement in the methodology is analogous to that used in the detection of C4 plant sugars in honey, by exploiting the internal isotopic correlations between the *δ*^13^C‰ value of the honey protein and sugars^5^. Candidate compounds for this would be citric acid CBNE *δ*^2^H values after conversion to aconitic acid to remove the exchangeable hydroxyl hydrogen.

## Methods

### Stable isotopic reference materials and chemical reagents

Stable isotope-certified reference materials (CRMs) USGS70, USGS71 (icosanoic acid methyl esters), USGS40, USGS41 (l-glutamic acids), were obtained from the United States Geological Survey, Reston Stable Isotope Laboratory. IAEA-CH-3 (cellulose) was obtained from the International Atomic Energy Agency (IAEA), Terrestrial Environment Laboratory. The *δ*^2^H and *δ*^13^C values assigned to these CRMs in this study for two-point normalisation to the VSMOW-SLAP and VPDB-LSVEC scale, respectively, were those reported by Schimmelmann et al.^34^ for USGS70 = −183.9‰_VSMOW-SLAP_ and USGS71 = −4.9‰_VSMOW-SLAP_, the Reston stable isotope laboratory^35,36^ for USGS40 = −26.39‰_VPDB-LSVEC_ and +37.63‰_VPDB-LSVEC_ and the IAEA Terrestrial Environment Laboratory^36^ for IAEA-CH-3 = –24.724 ‰_VPDB_. The reagents calcium hydroxide (96%), pyridine (99.5%), *N*-methyl-bis(trifluoroacetamide) (MBTFA) (≥97%), *n*-hexadecane (99.5%), d-(−)-fructose (99.0%), d-( + )-glucose (99.5%) and sucrose (99.5%), were purchased from the Sigma-Aldrich Chemical Company (Austria). The *n*-hexadecane obtained from the Sigma-Aldrich Chemical Company was calibrated as a quality-control in-house reference material and had an assigned value of *δ*^2^HVSMOW-SLAP of −89.09‰ _VSMOW-SLAP_.

### Authentic pineapple juice, retail pineapple juice and cane and beet sugar samples

Twenty authentic commercial production samples of single-strength pineapple juice (*n* = 3), pineapple puree (*n* = 2) and pineapple juice concentrate (*n* = 15) were obtained from the SGF international e.V. (a non-profit industrial association of the fruit juice sector with more than 600 members and participants in the Voluntary Control System of SGF from nearly 60 countries worldwide). The samples were collected in person by SGF representatives from industrial fruit juice production lines in countries that typically produce pineapple juice: Brazil (*n* = 4), China (*n* = 1), Costa Rica (*n* = 1), Indonesia (*n* = 2), Kenya (*n* = 1), Philippines (*n* = 1), South Africa (*n* = 1), Thailand (*n* = 8) and Vietnam (*n* = 1). An additional single-strength pineapple juice drink was purchased from a local retail outlet as an exemplary sample for method demonstration purposes. Samples were stored frozen at −18 °C prior to preparation. Pineapple juice concentrates and purees were diluted to 12 °Brix (~12% w/w of soluble solids) with Millipore water prior to sugar extraction, derivatisation and isotope analysis. Beet and cane sugar samples were obtained from retail outlets and their C3 and C4 identities confirmed through bulk carbon stable isotope analysis.

### Isolation of the pineapple juice sugars and derivatisation to their trifluoroacetate derivatives

A crude extract of the sugars present in the authentic pineapple juices was prepared by removal of pulp by centrifugation and the other major soluble solids, e.g. citric acid, by precipitation of their insoluble calcium salts following the procedure of Doner^37^. Briefly, a 30-cm^3^ aliquot of the defrosted juices was centrifuged at 10,397×*g* for 10 min. Calcium hydroxide was gradually added to the decanted supernatant in a separate beaker, with stirring, until the pH of the solution reached 8.5. Samples were then heated at 80 °C for ~15 min to allow for the formation of the calcium citrate precipitate. The precipitate was separated by filtration and the supernatant was deep-frozen in liquid nitrogen before being lyophilised overnight to yield the sugar fraction, which was stored in a light- protecting desiccator over silica gel. A portion of the residual sugars (25 mg) were derivatised with *N*-methyl-bis (trifluoroacetamide) (1 cm^3^) using pyridine as a solvent (1 cm^3^)^38^. This process removed exchangeable hydroxyl-hydrogen atoms and replaced them with trifluoroacetate (TFA) groups, which also makes the sugars sufficiently volatile for gas chromatographic separation and analysis.

### Hydrogen isotope analysis of the pineapple sugar trifluoroacetate derivatives

The major sugars in pineapple juice (sucrose, glucose and fructose) were separated as their TFA derivatives by gas chromatography and then passed into a capillary furnace containing chromium metal particles and silver wool maintained at 1200 °C. The other features of the GC-IRMS system (GC5-BioVisION, Elementar UK) used for the measurement of sugar-TFA derivatives are described in detail in Abrahim et al.^18^. In this system, the furnace retains carbon, oxygen and fluorine releasing hydrogen gas for determination of the mass distribution of its isotopologues (^2^H^1^H and ^1^H^1^H) by isotope ratio mass spectrometry. *δ*^2^H values were initially determined with respect to the hydrogen monitoring (cylinder) gas using the IRMS manufacturer’s proprietary software IonOS (Elementar UK) and then subsequently normalised to the VSMOW-SLAP scale using certified reference materials USGS70 and USGS71 (icosanoic acid methyl esters). A quality-control in-house reference of *n*-hexadecane was also analysed within each batch to monitor the performance of the normalisation process. The abundance ratio of deuterium to hydrogen isotopes (^2^H/^1^H) was expressed in the delta notation according to Eq. 3:

3 $$\delta^2 \rm{H}= R_{Sample}/R_{Reference}\;-\;1$$

### Carbon isotope analysis of pineapple juice sugars

The carbon isotope measurements were performed by combustion of the bulk sugar obtained after the fruit juice acid separation. A portion of the homogenised sugars (0.2 mg) was wrapped in a silver foil capsule (3 × 5 mm, Elementar, Germany) and combusted in an elemental analyzer Pyrocube (Elementar, Germany) coupled to a Bio-Vision isotope ratio mass spectrometer (Elementar UK). The quartz reactor tube was filled with tungstic oxide (tungsten(VI) oxide, WO_3_) and pure reduced copper wires in the reduction reactor maintained at 1020 and 850 °C, respectively. The *δ*^13^C values of the isolated bulk pineapple sugars were initially determined with respect to the carbon dioxide monitoring (cylinder) gas using the IRMS manufacturer’s proprietary software IonOS (Elementar UK) and then subsequently normalised to the V-PDB scale using certified reference materials USGS40 and USGS41 (l-glutamic acids). A quality-control in-house reference of IAEA-CH-3 cellulose was also analysed within each batch to monitor the performance of the normalisation process. The abundance ratio of carbon-13 to carbon-12 isotopes (^13^C/^12^C) was expressed in the delta notation according to Eq. 4:

4 $$\delta^{13}{\rm{C}}={R_{Sample}}/{R_{Reference}}-1$$

### Reporting summary

Further information on research design is available in the Nature Research Reporting Summary linked to this article.

## Supplementary information

Reporting Summary

Supplementary Information

## Data Availability

The isotopic data measured in this study are available under Supplementary Information.
