# Supplementary material for: Detection of exogenous sugars in pineapple juice using compound-specific stable hydrogen isotope analysis
Source: NPJ Sci Food. 2021 Apr 14;5:10. doi: 10.1038/s41538-021-00092-5 (PMC8046972; doi:10.1038/s41538-021-00092-5)
Supplement: Supplementary file 2 — Supplementary Information [file 41538_2021_92_MOESM2_ESM.pdf]

SUPPLEMENTARY INFORMATION for triplicate isotope analysis of authenticate pineapple juice.

				δ^{2}H‰			δ13C‰
	Sample#	Country/Product type		TFA Fructose	TFA Glucose	TFA Sucrose	Bulk
1	TFA SGF11037-18	Costa Rica/single strength	mean	36.78		7.51	-13.32
			SD	4.94		1.80	0.10
			min	29.61		4.66	-13.43
			max	42.82		9.42	-13.23
			mean peak height(nA)	0.85		4.32	
2	TFA SGF62060-16	Brazil juice concentrate	mean	2.49		24.24	-13.13
			SD	4.45		1.26	0.40
			min	-0.45		22.73	-13.56
			max	4.39		25.57	-12.76
			mean peak height(nA)	0.54		4.31	
3	TFA SGF36322-18	South Africa/juice concentrate	mean	2.70	1.20	16.00	-11.29
			SD	1.17	2.79	1.53	0.05
			min	1.90	-0.95	14.33	-11.33
			max	4.39	5.09	17.89	-11.25
			mean peak height(nA)	1.25	0.59	2.83	
4	TFA SGF28013-18	Thailand/juice concentrate	mean	4.58	0.73	10.55	-13.54
			SD	3.18	3.59	0.77	0.09
			min	1.46	-2.71	9.80	-13.62
			max	7.53	5.79	11.40	-13.45
			mean peak height(nA)	0.96	0.46	3.20	
5	TFA SGF62022-17	Brazil/juice concentrate	mean	-6.51		18.71	-14.28
			SD	2.87		0.58	0.16
			min	-10.01		17.98	-14.45
			max	-3.93		19.19	-14.14
			mean peak height(nA)	0.76		4.32	
6	TFA SGF28005-18	Thailand/juice concentrate	mean	11.17	15.66	31.24	-13.67
			SD	4.51	5.18	0.84	0.07
			min	4.49	9.68	30.35	-13.75
			max	14.39	22.26	32.38	-13.62
			mean peak height(nA)	1.13	0.53	3.58	
7	TFA SGF15253-18	Indonesia/juice concentrate	mean	24.45	47.41	2.61	-12.81
			SD	5.85	7.71	1.61	0.29
			min	15.75	37.08	0.40	-13.12
			max	28.19	55.69	4.10	-12.55
			mean peak height(nA)	0.94	0.42	3.18	
8	TFA SGF10035-17	Kenia/fresh fruit	mean	35.61	16.02		-11.31
			SD	2.01	2.93		0.01
			min	33.34	12.31		-11.32
			max	37.66	19.51		-11.29
			mean peak height(nA)	2.07	1.62		
9	TFA SGF11042-16	Indonesia/fresh fruit	mean	-2.32	-12.66		-14.53
			SD	1.54	3.67		
			min	-4.27	-18.26		
			max	-0.36	-8.44		
			mean peak height(nA)	1.62	0.85		
10	TFA SGF70117-16	Philipines/juice concentrate	mean	-33.79	-46.69	-24.98	-13.37
			SD	2.25	4.70	0.65	0.08
			min	-35.81	-50.19	-25.94	-13.42
			max	-30.57	-40.06	-24.53	-13.27
			mean peak height(nA)	0.92	0.43	3.72	
11	TFA SGF85012-17	China/single strength	mean	0.60	-9.75	12.70	-13.04
			SD	1.78	1.89	1.10	0.12
			min	-1.66	-12.13	11.30	-13.12
			max	3.05	-7.64	13.95	-12.96
			mean peak height(nA)	1.39	0.63	2.77	
12	TFA SGF78022-17	Vietnam/single strength	mean	-16.71	-28.25	-4.50	-14.08
			SD	0.61	3.55	0.98	
			min	-17.37	-33.78	-5.59	
			max	-15.96	-24.13	-3.30	
			mean peak hight (nA)	1.06	0.45	2.97	
13	TFA SGF70132-17	Indonesia/juice concentrate	mean	-25.91	-28.77	-9.88	-12.95
			SD	1.12	1.80	1.12	0.05
			min	-26.76	-30.47	-10.64	-13.00
			max	-24.64	-26.23	-8.59	-12.92
			mean peak hight (nA)	1.28	0.45	3.91	
14	TFA SGF78079-17	Thailand/juice concentrate	mean	-42.73		-23.51	-13.04
			SD	2.19		1.52	0.04
			min	-45.24		-24.95	-13.09
			max	-39.99		-21.73	-13.01
			mean peak hight (nA)	1.34		3.69	
15	TFA SGF 28022-18	Thailand/juice concentrate	mean	-18.77		-7.04	-14.87
			SD	3.84		1.44	
			min	-23.36		-8.30	
			max	-12.91		-4.87	
			mean peak hight (nA)	0.88		3.95	
16	TFA SGF22115-18	Brazil/juice concentrate	mean			14.17	-14.20
			SD			0.69	0.21
			min			13.47	-14.42
			max			14.86	-13.99
			mean peak hight (nA)			4.36	
17	TFA SGF28017-18	Thailand/juice concentrate	mean	-41.85		-16.87	-12.87
			SD	2.20		0.21	0.04
			min	-44.53		-17.10	-13.60
			max	-39.48		-16.60	-12.14
			mean peak hight (nA)	0.77		3.51	
18	TFA SGF72008-17	Brazil/Puree	mean	-11.05		-8.13	-12.64
			SD	1.48		2.16	0.28
			min	-12.39		-9.66	-12.88
			max	-9.54		-5.02	-12.33
			mean peak hight (nA)	0.58		4.63	
19	TFA SGF78015-17	Thailand/juice concentrate	mean	-37.49		-18.93	-13.72
			SD	1.26		0.57	0.04
			min	-38.47		-19.70	-13.74
			max	-35.71		-18.40	-13.69
			mean peak hight (nA)	0.98		4.03	
20	TFA SGF78023-16	Thailand/juice concentrate	mean	-30.23		5.23	-13.38
			SD	2.46		0.39	0.07
			min	-33.83		4.87	-13.46
			max	-28.56		5.64	-13.33
			mean peak hight (nA)	0.71		3.48	
21	TFA SGF70176-17	Kenia/puree	mean	17.87		17.32	-12.52
			SD	4.77		0.63	0.02
			min	12.78		16.37	-12.54
			max	22.22		17.72	-12.49
			mean peak hight (nA)	0.73		3.56	
22	TFA SGF78052-17	Thailand/juice concentrate	mean	-50.64		-39.63	-13.03
			SD	1.88		1.30	0.04
			min	-52.81		-40.36	-13.06
			max	-48.77		-37.70	-12.98
			mean peak hight (nA)	0.89		3.40	
